# Cina—Rheumatism

**Published:** 1881-08

**Authors:** William A. Hawley

**Affiliations:** Syracuse, N. Y.


### INFLAMMATORY RHEUMATISM.—CINA.

William A. Hawley, M.D., Syracuse, N.Y.

On the 31st of March, 1881, I found Mabel—aged ten years, of light complexion, but dark hair and eyes, after three days of illness, which began with lameness and soreness of the left arm and elbow, and passed the next day to the right knee, and then to the ankles—lying on her back, with legs extended, hands by her side, ankles considerably swollen and hot, with an oval patch of redness about two and a half by five inches covering the internal malleoli.

She was afraid to move a muscle anywhere, on account of great pain, but was quite easy so long as she was motionless. If either foot was moved in the least, she screamed with pain. There were pain and soreness, but no swelling nor redness, in both groins, hips and down the thighs on motion; cheeks very red; dark under the eyes; *very white and bluish about the mouth;* tongue covered with a thin white coat; great thirst; no appetite; fetid breath; skin moist; sleep broken, always waking with a start and incoherent talking; urine high colored; and a desire for stool, which she could not void, because of pain resulting from the necessary motion,

The first remedy I thought of was *Cina.* But has that drug any rheumatic symptoms? I could not remember any. However, thinking not to spoil the case by guessing, and giving a remedy not indicated, I gave her *Sac. lac.* in water, once in two hours. I find, under *Cina* in the symptomen Codex, "general symptoms," painful sensitiveness in every limb of the body when moving or touching it, but no heat, swelling, nor redness of any part. As I could not find any other drug (although I studied as closely as I could those ordinarily prescribed for rheumatism) that so well covered the totality of the case, and finding the child just in the same condition and position the next morning, I gave a few pellets of *Cina* 200th, dissolved in a gill of water, a teaspoonful once in three hours. The third morning her salutation was: "Oh, doctor! my ankles are both well, and I can move them so!" bending her ankles up and down as she spoke. How about the hips and groins? "They hurt me yet." She was still lying on her back, as when I first saw her, but, on testing her a little, I found that the legs could be moved passively. I helped her to turn on her side, and continued the remedy. The fourth visit found her sitting up, the tongue clean; appetite returned; lame ness all gone, and color of face normal. The next day her father reported that she was up and about the house "all right."

I have written this because it is to me additional proof that conformity to the law of the similars, the single remedy, and the minimum dose will cure, regardless of names. Have others seen such inflammatory symptoms so disappear under the action of *Cina?*

[Since the above was received, Dr. Hawley, in a letter to us, describes the difficulty he had in finding the symptom which led to giving *Cina*, and the following verification of it]:

Since writing the above, I have had a confirmation of my idea of the characteristic peculiarity of that symptom (*painful sensation in every limb of the body when moving or touching it*). I was called to see a case, with my friend, Dr. S——, a girl, ten years old, sick for three or four days. The most troublesome symptom was paroxysmal pains of a tearing character through the right side of face and down the back, which she wanted rubbed all the time. She constantly kept up the motion of swallowing. Regarding that as the key-note, I advised *Cina*. Dr. S. was giving *Merc. sol.* 200th. As he thought the case doing well, he did not change, but continued the *Merc.* until the next morning, when he thought *Rhus.* better indicated, and gave it. In the evening she was not relieved, and he found so much soreness of arms that she objected to having them moved or touched, which so reminded him of my case, that he gave her *Cina*. The child immediately improved.

